# White Matter Biomarkers Associated with Motor Change in Individuals with Stroke: A Continuous Theta Burst Stimulation Study

**DOI:** 10.1155/2019/7092496

**Published:** 2019-02-04

**Authors:** K. P. Wadden, S. Peters, M. R. Borich, J. L. Neva, K. S. Hayward, C. S. Mang, N. J. Snow, K. E. Brown, T. S. Woodward, S. K. Meehan, L. A. Boyd

**Affiliations:** ^1^Faculty of Medicine, Memorial University of Newfoundland, St. John's, Canada; ^2^University of British Columbia, Department of Physical Therapy, Vancouver, Canada; ^3^Division of Physical Therapy, Department of Rehabilitation Medicine, Emory University School of Medicine, Atlanta, USA; ^4^Stroke Division, Florey Institute of Neuroscience and Mental Health, University of Melbourne, Melbourne, VIC 3084, Australia; ^5^NHMRC Centre of Research Excellence in Stroke Rehabilitation and Brain Recovery, Australia; ^6^University of Regina, Faculty of Kinesiology and Health Studies, Regina, Canada; ^7^Department of Clinical and Movement Neurosciences, University College London, London, UK; ^8^University of British Columbia, Department of Psychiatry, Vancouver, BC, Canada; ^9^BC Mental Health and Addictions Research Institute, Vancouver, BC, Canada; ^10^Department of Kinesiology, University of Waterloo, Waterloo, Canada; ^11^University of British Columbia, Centre for Brain Health, Vancouver, Canada

## Abstract

Continuous theta burst stimulation (cTBS) is a form of noninvasive repetitive brain stimulation that, when delivered over the contralesional hemisphere, can influence the excitability of the ipsilesional hemisphere in individuals with stroke. cTBS applied prior to skilled motor practice interventions may augment motor learning; however, there is a high degree of variability in individual response to this intervention. The main objective of the present study was to assess white matter biomarkers of response to cTBS paired with skilled motor practice in individuals with chronic stroke. We tested the effects of stimulation of the contralesional hemisphere at the site of the primary motor cortex (M1c) or primary somatosensory cortex (S1c) and a third group who received sham stimulation. Within each stimulation group, individuals were categorized into responders or nonresponders based on their capacity for motor skill change. Baseline diffusion tensor imaging (DTI) indexed the underlying white matter microstructure of a previously known motor learning network, named the constrained motor connectome (CMC), as well as the corticospinal tract (CST) of lesioned and nonlesioned hemispheres. Across practice, there were no differential group effects. However, when categorized as responders vs. nonresponders using change in motor behaviour, we demonstrated a significant difference in CMC microstructural properties (as measured by fractional anisotropy (FA)) for individuals in M1c and S1c groups. There were no significant differences between responders and nonresponders in clinical baseline measures or microstructural properties (FA) in the CST. The present study identifies a white matter biomarker, which extends beyond the CST, advancing our understanding of the importance of white matter networks for motor after stroke.

## 1. Introduction

Incomplete recovery of movement from stroke has led to interest in adjunct interventions. One such intervention is noninvasive brain stimulation that, when paired with therapy, may augment the effects of rehabilitation [1–3]. Continuous theta burst stimulation (cTBS) is a patterned form of repetitive transcranial magnetic stimulation (rTMS) that can suppress excitability of the primary motor cortex (M1) [4]. Following a stroke, there is evidence that the contralesional cortex exerts increased inhibition on the ipsilesional cortex through interhemispheric signaling [5]. In individuals with stroke, cTBS applied over the contralesional hemisphere may modulate interhemispheric imbalances to the ipsilesional hemisphere [6]. However, a large degree of individual variability in response to repetitive noninvasive brain stimulation techniques has been observed [2, 7]. As a result, there is an important focus on investigating biological markers (“biomarkers”) that characterize “responders” and “nonresponders” of noninvasive brain stimulation [2, 7].

To date, most research has focused on regional white matter (WM) tracts [2, 7] as biomarkers of recovery after stroke. However, recovery from stroke involves a network of bihemispheric pathways that extend between the contralesional and ipsilesional motor cortices, secondary motor areas, and ipsilesional cerebellum [8]. In the current work, we hypothesized that characterizing a specialized WM motor network associated with motor learning would explain response to cTBS paired with motor skill practice in individuals with chronic stroke [9]. We employed functional magnetic resonance imaging- (fMRI-) guided tractography to constrain WM connections associated with our previously identified gray matter (GM) motor learning network associated with motor learning in healthy individuals [9]. We named the resultant network the “constrained motor connectome” (CMC) and hypothesized that individual capacity for motor learning–related change following cTBS paired with motor skill practice would relate to residual integrity in the CMC [9].

The primary motor and somatosensory cortices (M1 and S1, respectively) are two brain areas that support motor recovery [10]. Hyperexcitability from both the contralesional M1 and S1 (M1c, S1c) correlates with reduced poststroke motor function [11]. Yet, few studies have extended noninvasive brain stimulation sites beyond contralesional M1 [1, 3]. Meehan et al. [3] compared the effects of cTBS over contralesional M1 versus S1 paired with motor practice and found comparable improvements in movement time during motor skill practice. Regardless of stimulation site, both M1 and S1 stimulation groups showed larger amounts of motor learning–related change compared to sham stimulation paired with motor skill practice [3]. During repetitive TMS over M1, stimulation spread of 2-3 cm over the cortex from the centre of coil [12] as well as strong connections between M1 and S1 [13] have been observed. Therefore, based on similar effects of cTBS over M1c and to S1c previously observed on movement time [3], and the proximity of the locus of stimulation sites (i.e., 2 cm apart), we hypothesized that improvements in motor performance would be *similar* for the M1c and S1c stimulation.

The overarching objective of the current study was to test whether a new brain-based biomarker, termed the CMC, could identify the capacity to respond to cTBS over M1c/S1c paired with motor skill practice. We first studied the effect of cTBS over M1c or S1c paired with motor skill practice on motor learning. We discovered highly variable responses to this intervention. We next categorized individuals in the M1c and S1c groups into responders or nonresponders based on the extent of their behavioural change. Given a lack of difference between individuals in the M1c and S1c groups, and to maintain power to consider responder status, we next combined responders from the two stimulation groups (M1c and S1c) into a sensorimotor (SM) cTBS group; the same was done for the nonresponders. We hypothesized that the integrity of the CMC [9] would explain response to cTBS over the SM paired with motor skill practice.

## 2. Methods

### 2.1. Participants

Twenty-eight individuals (mean age = 63.0 years; standard deviation (SD) = 12.88 years; 7 females) who demonstrated chronic stroke-related unilateral upper limb deficits were recruited. All experimental sessions were completed at the University of British Columbia (UBC). Ethical approval was granted from the Clinical Research Ethics Board of UBC. All participants provided written informed consent in accordance with the Declaration of Helsinki.

Inclusion criteria were (1) chronic cortical or subcortical stroke (≥6 months ago), (2) upper-extremity Fugl-Meyer (UE-FM) motor impairment score greater than or equal to 15, and (3) a Montreal Cognitive Assessment (MoCA) score greater than or equal to 26 [14]. Exclusion criteria were (1) history of seizure/epilepsy, head trauma, a major psychiatric diagnosis, neurodegenerative disorder, or substance abuse; (2) taking any gamma-aminobutyric acid (GABA) ergic, N-methyl-D-aspartate (NMDA) receptor antagonist, or other drug known to influence the neural receptors that facilitate neuroplasticity; or (3) contraindication to transcranial magnetic stimulation (TMS) or magnetic resonance imaging (MRI).

### 2.2. Experimental Design

**Figure 1:**
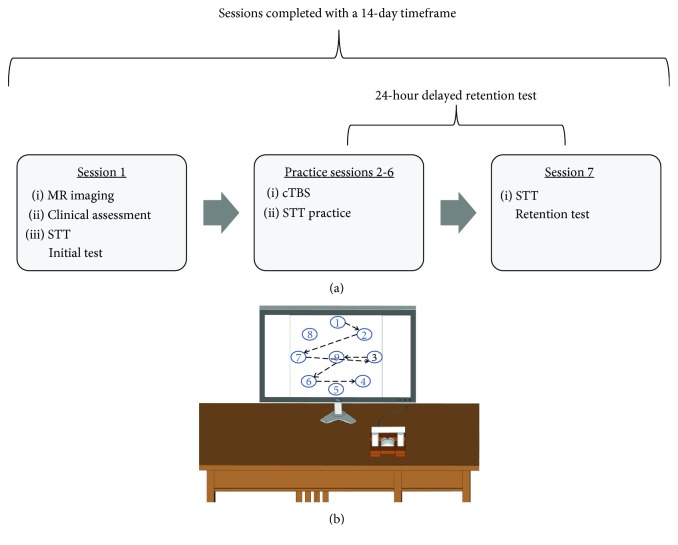
Experiment design and apparatus. (a) Experimental design and (b) serial tracking task (STT) apparatus and target locations.

Participants were pseudo-randomly assigned to one of three stimulation groups: (1) M1c cTBS (contralesional primary motor cortex), (2) S1c cTBS (contralesional primary somatosensory cortex), or (3) sham cTBS over contralesional M1 (Figures 1(a) and 1(b)). Pseudo-randomization was accomplished using a custom computer program that assigned individuals to a stimulation group while accounting for age, sex, and Fugl-Meyer (FM) score to ensure even distribution (M1c, *n* = 9; S1c, *n* = 11; and sham, *n* = 8). The experimental protocol consisted of seven sessions over 14 days. There were never more than two days between sessions, and the retention session was performed within 24 hours of the last practice session (Figure 1). In session 1, participants underwent MR scanning, clinical assessments of motor function and impairment (Wolf Motor Function Test (WMFT) and FM, respectively), and baseline performance on the experimental motor learning task, the serial targeting task (STT). During sessions 2 to 6, participants received cTBS over the contralesional hemisphere according to the stimulation group (M1c, S1c, and sham) immediately before STT practice. In session 7, a no-cTBS STT retention test was employed to assess motor learning.

In session 1, to assess upper-extremity motor function, the WMFT was performed by a licensed physical therapist. Mean performance time to complete 15 items of the WMFT with the paretic and nonparetic arms was determined. Participants' WMFT rate was calculated to determine how many times an individual could complete the task continuously for 60 seconds (60 seconds divided by the performance time); if an individual could not perform the task in 120 seconds, a score of 0 was given [15]. In addition, individuals' physical impairment level was assessed via the upper-extremity (UE) FM scale (range 0-66; lower scores denote less paretic arm function). Participant characteristics are shown in Table 1.

### 2.3. Serial Targeting Task

Participants performed the STT seated. The paretic hand (pronated) was used to grasp a computer mouse (housed in a custom frame) to control the movements of an on-screen cursor (Wheel Mouse Optical, Microsoft Corporation, Redmond, Washington, USE). Individuals were instructed to move the cursor as quickly and accurately as possible to a series of sequentially appearing targets. The location of the participant's hand in space was occluded by an opaque surface affixed above the hand. Embedded within the series of targets was a repeated six-element sequence that was flanked by a six-element random sequence. The participant performed four blocks of the STT during each practice session (2 to 6). Each block was comprised of nine random sequences that each contained six movements each, and eight repeated sequences that also contained six movements each. Participants performed one block of the STT in sessions 1 (baseline) and 7 (retention) to index motor learning [3].

### 2.4. Exponential Curve Fitting

The primary dependent measure was response total time (RTT). Participants' RTT was calculated as the time to initiate movement plus movement time. The sum RTT for all six movements within the repeated and random sequences was calculated separately. The RTT for repeated and random sequences in each block across the seven sessions (baseline, session 1; practice, sessions 2 to 6; and retention, session 7) of task performance for each participant was subsequently fit to separate exponential functions using the following equation [16, 17]:

(1) $$\begin{array}{c} E\left({\text{RTT}}_{N}\right)=A+{Be}^{-\alpha N}. \end{array}$$

*E*(RTT_*N*_) is the expected value of RTT on practice trial *N*. *A* is the expected values of RTT after practice has been completed (asymptote parameter). *B* is the expected change in RTT from session 1 to session 7 (change score parameter). Alpha (*α*) is the exponential motor skill acquisition rate parameter [18]. Our primary outcome measure was *B*, which reflects an individual's capacity for motor change. A custom MATLAB (Version R2013b, The MathWorks Inc., Natick, Massachusetts, USA) script was used for all analyses.

#### 2.4.1. Motor Practice Responder versus Nonresponder

The *B* score was used to differentiate between responders and nonresponders. A positive *B* score reflects an individual's capacity for motor learning based on the performance plateau prediction, while a negative *B* score indicates an absence of motor learning–related change [19].

### 2.5. Transcranial Magnetic Stimulation Procedures

All participants were screened for contraindications to rTMS [20]. TMS was performed using Magstim Rapid^2^ and Plus^1^ magnetic stimulators and a 70 mm diameter air-cooled figure-of-eight coil (Magstim Co. Ltd., Whitland, Carmarthenshire, UK) on sessions 2 to 6. During all TMS procedures, participants were seated in a reclining chair with their hands placed in a relaxed position (elbow at 180 degrees flexion, forearm pronated). For all stimulation, coil positioning was continuously monitored using a Brainsight™ neuronavigation system, which displayed each individual's T1-weighted MRI. The participants' motor hotspot (M1c) and resting motor threshold (RMT) were determined as the site that evoked a measurable MEP greater than or equal to 50 *μ*V peak-to-peak for 5 out of ten trials in the extensor carpi radialis (ECR) muscle, at the lowest stimulus intensity at rest. After identifying the ECR motor hotspot, the active motor threshold (AMT) was determined as the lowest intensity to evoke a 200 *μ*V MEP in at least five out of 10 TMS stimuli [21], while participants maintained a 20% maximal isometric voluntary hand grip contraction. Thereafter, cTBS was then applied with the participant at rest in the theta burst pattern of stimulation: three stimuli delivered at 50 Hz, grouped and delivered at 5 Hz, in continuous blocks for a total of 600 stimuli over 40 seconds [4] at an intensity of 80% AMT. Sham stimulation was performed with a dedicated coil that looked and sounded like active stimulation but did neither mimic the cutaneous sensation provided during active stimulation nor induce any current in the underlying cortex (Magstim Company Ltd.). Continuous TBS was delivered over the determined M1c “hotspot” or two cm posterior to this location for S1c, consistent with previous work [1, 3], while sham was delivered over the M1c hotspot. Following cTBS completion on sessions 2 to 6, after a five-minute break, participants completed motor practice of the STT.

### 2.6. Magnetic Resonance Imaging Protocol

MR acquisition was conducted at the UBC MRI Research Centre on a Philips Achieva 3.0 T whole-body MRI scanner (Phillips Healthcare, Andover, Massachusetts) using an eight-channel sensitivity encoding head coil (SENSE factor = 2.4) and parallel imaging.

#### 2.6.1. Anatomical Scan

A high-resolution T1-weighted anatomical scan (TR = 7.47 ms, TE = 3.65 ms, flip angle *Ɵ* = 6°, FOV = 256 × 256 mm, 160 slices, and 1 mm^3^ isotropic voxel) was collected.

#### 2.6.2. Diffusion-Weighted Magnetic Resonance Imaging (DW-MRI)

One high-angular resolution diffusion imaging (HARDI) scan was performed using a single-shot echo-planar imaging (EPI) sequence (TR = 7096 ms, TE = 60 ms, FOV = 224 × 224 mm, 70 slices, and voxel dimensions = 2.2 × 2.2 × 2.2 mm^3^). Diffusion weighting was applied across 60 independent noncollinear orientations (*b* = 700 s/mm^2^), along with five unweighted images (*b* = 0 s/mm^2^). Diffusion-weighted images (DWIs) were corrected for motion and distortion using the software package ExploreDTI v4.2.2 (http://www.exploredti.com; [22]). During motion and distortion correction, signal intensity was modulated and the *b*-matrix was rotated [22].

#### 2.6.3. Corticospinal Tract (CST)

**Figure 2:**
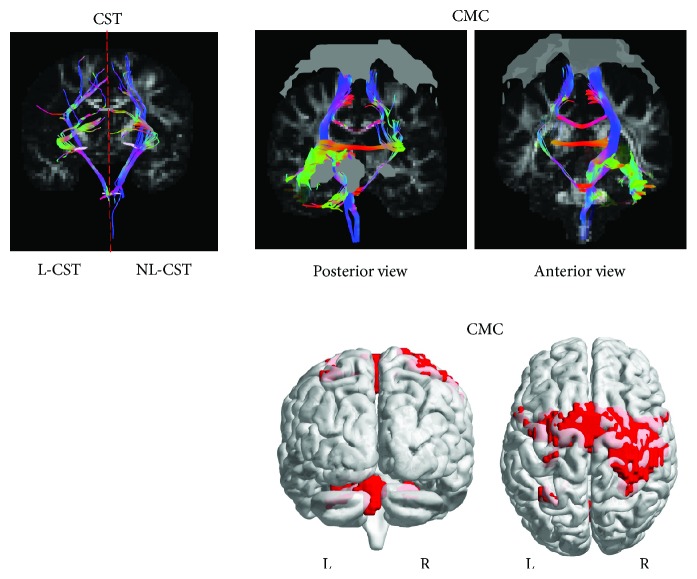
(a, b_1_, and b_2_) Diffusion tensor imaging. Fractional anisotropy (FA) maps were created for individuals, followed by whole-brain tractography. This is an example of a single subject with a left hemispheric lesion. (a) Regions of interest (ROIs) for the nonlesioned (NL) and lesioned (L) corticospinal tracts (CST) were manually drawn on the native DW image, followed by tractography. The first cross-sectional ROI for the CST was delineated bilaterally (NL-CST and L-CST) in the axial plane [52]. First, a “SEED” ROI was constructed around the PLIC at the level of the anterior commissure [53]. Second, a logical “AND” ROI was constructed around the CST at the level of the mid-pons [54]. The “AND” function constrained the reconstruction to fibers passing through both the “SEED” and “AND” ROI. (b_1_) The functional motor network mask (gray ROIs) was extracted and overlaid on the DW MNI image, followed by tractography of the CMC (posterior and anterior views). (b_2_) The motor network mask (represented in red) was overlaid on the diffusion-weighted image to create the constrained motor connectome (CMC).

Diffusion-weighted images were analyzed using ExploreDTI. All images remained in native space. At each voxel of the CST, constrained spherical deconvolution-based deterministic whole-brain fiber tractography was initiated using the following parameters: seedpoint resolution of 2 mm^3^, 0.2 mm step size, maximum turning angle greater than 40°, and fiber length range of 50 to 500 mm [23]. We used a constrained spherical deconvolution approach to analyze known pathways of the corticospinal tract (CST), to ensure the inclusion of all tracts where we could manually control their inclusion and exclusion and reduce false positives (see Figure 2 for methods).

#### 2.6.4. Constrained Motor Connectome (CMC)

Data from the CMC were first transformed into Montreal Neurological Institute Space. DTI analyses were completed using ExploreDTI. DTI is recommended when long-range axonal connectivity is of interest [24]. Therefore, for the CMC analysis we used the more conservative DTI tractography approach as we had no a priori guidelines to identify tracts within this motor network. At each voxel of the CMC, DTI-based deterministic whole-brain fiber tractography was initiated using the following parameters: seedpoint resolution of 2 mm^3^, FA threshold 0.2, 0.2 mm step size, maximum turning angle greater than 40 °, and fiber length range of 50 to 500 mm. FA values, the most commonly reported measure of white matter microstructural integrity after stroke, were extracted from reconstructed tracts and used for statistical analyses [25]. FA is a quantitative, unit-less measure of diffusion behaviour of water in the brain; a value of zero indicates diffusion of water as isotropic, and a value of one specifies a preferred direction of diffusion along one axis [26].

We used a previously defined functional motor network associated with motor learning in healthy individuals [9]. Selected binary masks of cortical GM clusters of activation were used as ROIs for WM tractography. Each of the four clusters encompassed multiple brain regions. Cluster one included the primary somatosensory cortex, motor cortex, precentral gyrus, bilaterally, and the right intraparietal, superior parietal, and inferior parietal cortices. Cluster two included lobule V, VI, VIIIa, and VIIIb of the cerebellum, bilaterally. Cluster three included right lobule VI and VIIa Crus of the cerebellum. Cluster four included left intraparietal and superior parietal cortices (Table 2 and Figure 2(b), b1). The GM cortical clusters from the functional motor network were derived from a whole-brain connectivity analysis in MNI space, allowing for the clusters of activation from the fMRI connectivity analysis to be overlaid on the DW images converted to MNI space. The functional motor network ROIs were used to isolate the underlying WM fiber tracts of the CMC (Figure 2(b), b1).

### 2.7. Statistical Analyses

Four main investigative steps were performed. These included (1) determining the effect of stimulation group (M1c, S1c, and sham) on motor sequence learning, (2) separating individuals into motor practice responders and nonresponders using the B score, (3) assessing differences in demographic and clinical measures between motor practice responders and nonresponders for the SMc-cTBS group, and (4) testing for differences in WM biomarkers between motor practice responders and nonresponders for the SMc-cTBS group.

### 2.8. Differences in Motor Sequence Learning between cTBS Groups in Individuals with Stroke during Practice and Retention

#### 2.8.1. Baseline Performance

Session 1 (baseline) motor performance on the random and repeated sequences was evaluated with a two-factor GROUP (M1c, S1c, and sham) × SEQUENCE (repeated, random) mixed-model analysis of variance (ANOVA) with mean RTT as the dependent variable.

#### 2.8.2. Practice Performance

Performance of the repeated and random sequences during cTBS paired with motor skill practice (sessions 2 to 6) was examined using a three-factor GROUP (M1c, S1c, and sham) × SEQUENCE (repeated, random) × SESSION (sessions 2 to 6) mixed-model ANOVA with mean RTT as the dependent variable. There were two missing data points for practice days for two participants in the sham group (*n* = 6).

#### 2.8.3. Retention Performance

To assess motor sequence learning at retention (session 7), a two-factor GROUP (M1c, S1c, and sham) × SEQUENCE (repeated, random) mixed-model ANOVA was performed with mean RTT as the dependent variable.

### 2.9. Effect of cTBS on Motor Sequence Learning for Motor Practice Responders

**Figure 3:**
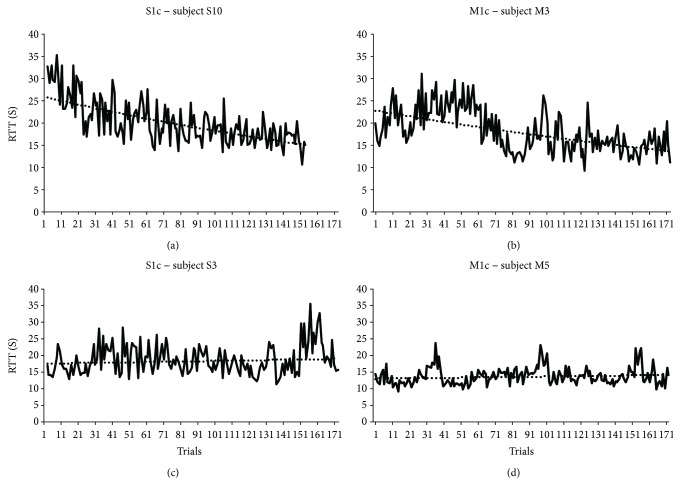
(a, b, c, and d) Active cTBS motor practice responder and nonresponders. The top panel represents motor practice responders (positive *B* score) following contralesional cTBS, delivered over the primary somatosensory cortex (S1c; B score = 14.27; (a)) or primary motor cortex (M1c; *B* score = 12.30; (b)), paired with motor skill practice. The bottom panel represents motor practice nonresponders (negative b score) following contralesional cTBS, delivered over the primary somatosensory cortex (S1c; *B* score = −4.35; (c)) or primary motor cortex (M1c; *B* score = −1.76; (d)) paired with motor skill practice.

Motor practice responders were identified as individuals who demonstrated a positive *B* (*B* > 0) score for repeated sequences, and nonresponders were identified as individuals who demonstrated a negative *B* score (*B* < 0) (see Figure 3 for subject-specific examples). We tested the effect of stimulation (M1c, S1c, and sham) on performance for the *motor practice responder group*. Based on our hypothesis that there would be similarities between the effect of cTBS over M1c and S1c on RTT, we performed a planned independent *t*-test on the *B* score between M1c and S1c groups for motor practice responders.

We had an a priori hypothesis that groups receiving inhibitory stimulation over the contralesional hemisphere (M1c, S1c groups) would demonstrate *greater* improvements in motor performance compared to individuals receiving sham stimulation (sham group) [3, 27–31]. To evaluate the effects of receiving stimulation, M1c and S1c groups were combined into a contralesional sensorimotor (SMc-cTBS group) group. To evaluate the effects of receiving active cTBS stimulation over SMc compared to sham stimulation, we performed a planned one-tailed independent *t*-test on *B* score between SMc-cTBS and sham groups for motor practice responders.

#### 2.9.1. Clinical Measures for Motor Practice Responders and Nonresponders

**Figure 4:**
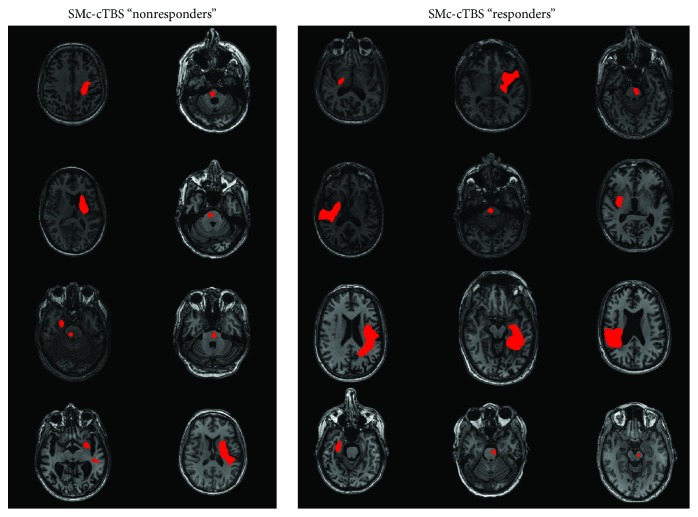
Lesion figure for M1c and S1c (sensorimotor- [SM]-) cTBS motor practice responders versus nonresponders.

To investigate whether differences existed in clinical measures between responders and nonresponders, we conducted independent group *t*-tests to assess differences in demographic and clinical characteristics. Demographic and clinical dependent variables included age, poststroke duration, UE-FM score, and paretic WMFT rate. Fisher's exact test was used to assess differences between motor practice responders and nonresponders in stroke location (cortical, C; subcortical, SC) for these individuals (Table 3; see Figure 4 for stroke locations).

#### 2.9.2. WM Tractography for Motor Practice Responders and Nonresponders

A multivariate analysis of variance (MANOVA) was used to assess differences in WM-FA from the CMC and NL- and L-CST between motor practice responders and nonresponders within the SMc-cTBS group (S1c, M1c). The dependent variables for the MANOVA were FA values for each ROI (CMC, and NL- and L-CST). *Post hoc* univariate ANOVAs were performed on significant (*p* ≤ 0.05) MANOVAs.

In the event of a violation of sphericity (significant Mauchly's test, *p* ≤ 0.05), the Greenhouse-Geisser correction was applied. Levene's test for equality of variances was used to test for homogeneity of variance, and degrees of freedom were adjusted when the test was significant (*p* ≤ 0.05). The 95% confidence intervals (CIs) of the mean difference (MD) were used to describe the effect of stimulation on improvements in motor performance (*B* score). Effect sizes were reported as partial eta-squared (*η*_*ρ*_^2^) where 0.01 is considered a relatively small effect, 0.06 moderate, and more than 0.14 a large effect [32]. Significance level for all statistical tests was set at *p* ≤ 0.05, and post hoc tests, Bonferroni-corrected for multiple comparisons, were conducted when appropriate. Data are presented in the text as mean (M) plus or minus SD or standard error (SE). All statistical procedures were conducted using SPSS software (Version 21.0, IBM Corporation, Armonk, New York).

## 3. Results

### 3.1. Repeated versus Random Sequence Performance and Learning between cTBS Stimulation Groups

#### 3.1.1. Baseline Performance

**Figure 5:**
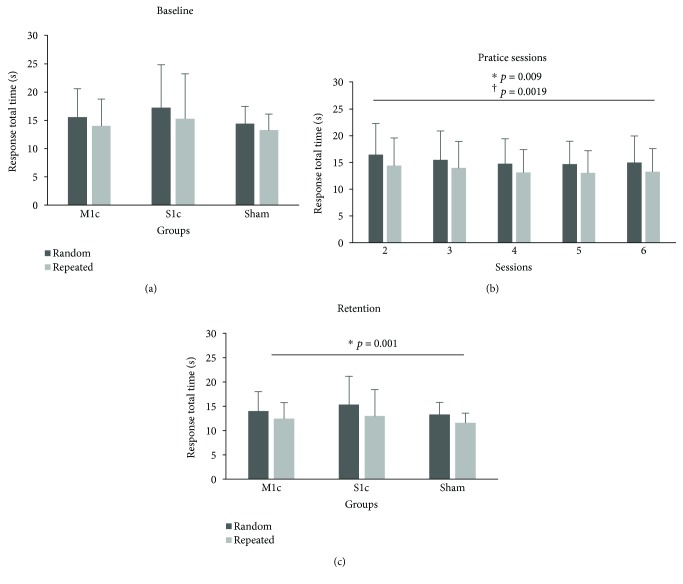
(a, b, and c) Mean response total time (RTT) for repeated and random sequences. Stimulation groups (M1c, S1c, and sham) demonstrated similar performances for repeated and random sequences on session 1 (baseline), 2 to 6, and 7 (retention). (a) All groups demonstrated initial faster RTTs for repeated compared to random sequence performance. (b) Collapsed across groups, all individuals demonstrated faster performance for repeated compared to random sequences across the five days of practice: *F*_(1, 25)_ = 29.94, *p* < 0.001. (c) All groups demonstrated faster RTTs for repeated compared to random sequence during retention performance: *F*_(1, 23)_ = 19.63, *p* < 0.001. Error bars are SD of the mean.

During initial STT performance (session 1), the random and repeated sequences trended towards, but were not, statistically different (*F*_(1, 25)_ = 4.09, *p* = 0.054, *η*_*ρ*_^2^ = 0.141; Figure 5(a)). There was no baseline difference in performance level between groups (M1c, S1c, and sham) as shown by the lack of significant main effect of GROUP (M1c, S1c, and sham) (*F*_(2, 23)_ = 0.48, *p* = 0.63, *η*_*ρ*_^2^ = 0.037). Additionally, there was no significant GROUP × SEQUENCE interaction in session 1 (*F*_(2, 23)_ = 0.084, *p* = 0.92, *η*_*ρ*_^2^ = 0.007).

#### 3.1.2. Practice Performance

All groups (M1c, S1c, and sham) demonstrated improved motor performance on the STT, evidenced by an observed decrease in RTT across sessions 2 to 6 and a significant main effect of SESSIONS (*F*_(2.55,58.71)_ = 4.51, *p* = 0.009, *η*_*ρ*_^2^ = 0.164; Figure 5(b)). Mauchly's test indicated that the assumption of sphericity had been violated (*χ*^2^(9) = 27.63, *p* = 0.001); therefore, degrees of freedom were corrected using Greenhouse-Geisser estimate of sphericity (*ε* = 0.638) for main effect of SESSIONS. In addition, individuals showed superior repeated (*M* = 13.88, SE = 0.90) compared to random sequence (*M* = 15.59, SE = 0.99) performance across practice sessions, as revealed by the significant main effect of SEQUENCE (*F*_(1, 23)_ = 19.63, *p* = 0.0019, *η*_*ρ*_^2^ = 0.46). However, there was no main effect of GROUP (M1c, S1c, and sham) (*F*_(2, 23)_ = 0.066, *p* = 0.94, *η*_*ρ*_^2^ = 0.006), no significant interaction for SEQUENCE × SESSION (*F*_(4, 92)_ = 0.37, *p* = 0.83, *η*_*ρ*_^2^ = 0.016), or for GROUP × SEQUENCE (*F*_(2, 23)_ = 0.29, *p* = 0.749, *η*_*ρ*_^2^ = 0.025).

#### 3.1.3. Retention Test Performance

Motor learning–related change was shown by a main effect of SEQUENCE that confirmed all groups were faster for repeated (*M* = 12.34, SE = 0.772) compared to random sequence (*M* = 14.20, SE = 0.861) at retention (*F*_(1, 25)_ = 29.94, *p* < 0.001, *η*_*ρ*_^2^ = 0.55; Figure 5(c)). However, the main effect of GROUP (M1c, S1c, and sham) (*F*_(2, 25)_ = 0.38, *p* = 0.68, *η*_*ρ*_^2^ = 0.030) and the GROUP × SEQUENCE interaction (*F*_(2, 25)_ = 0.64, *p* = 0.53, *η*_*ρ*_^2^ = 0.049) were not significant.

#### 3.1.4. Motor Practice Responders

**Figure 6:**
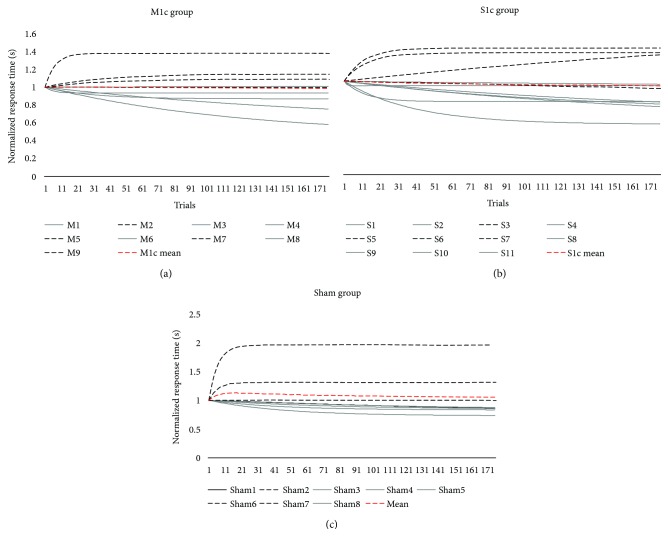
(a, b, and c) Motor skill acquisition curves for S1c, M1c, and sham stimulation groups. Single-subject normalized motor skill exponential curves for active cTBS stimulation groups (top panel) delivered over the contralesional primary somatosensory (S1c; (a)) and contralesional primary motor cortex (M1c; (b)) and for the sham stimulation group (bottom panel; (c)). Dotted black line indicates individuals with negative *B* scores and identified as motor skill nonresponders. Solid grey line indicates individuals with positive *B* scores and identified as motor practice responders. Dashed red line represents the mean motor skill acquisition curve for each stimulation group.

Overall, there were 17 motor practice responders and 11 nonresponders, as indicated by a positive *B* score for responders and a negative *B* score for nonresponders (M1c: 5 responders, 4 nonresponders; S1c: 7 responders, 4 nonresponders; and sham: 5 responders, 3 nonresponders) (see Figure 6 for normalized *B* score; normalization factor of *A* + *B*; *A* = asymptote value; *B* = change score). For motor practice responders, the first planned comparison showed no significant difference for *B* score between M1c (*M* = 5.87, SD = 5.154) and S1c (*M* = 5.66, SD = 4.922) groups for the performance of the repeated sequence (*t*_(10)_ = 0.074, *p* = 0.94, 95% CI [−6.32, 6.76]).

Following the amalgamation of M1c and S1c groups into the SMc-cTBS group, the second planned comparison demonstrated a significantly larger improvement in motor performance (*B* score) for the SMc-cTBS group (*M* = 5.74, SD = 4.784) compared to the sham group (*M* = 3.06, SD = 1.146), *t*_(13.54)_ = 1.82, *p* = 0.045, 95% CI [−0.48, 5.86].

#### 3.1.5. Clinical Baseline Measures for the SMc cTBS Group

In the combined SMc-cTBS group, 12 of 20 participants responded positively to cTBS paired with motor skill training, as evidenced by a positive *B* score. When considering the individuals in the SMc-cTBS group, independent group *t*-tests and a Fisher's exact test (binary data for stroke location [C: 1; SC: 0]) demonstrated no significant differences in demographic (age: *t*_(18)_ = 0.08; *p* = 0.93) or clinical characteristics (stroke location: *p* = 0.64; PSD: *t*_(18)_ = 0.70, *p* = 0.49; UE-FM: *t*_(18)_ = 0.25, *p* = 0.81; and paretic WMFT rate: *t*_(18)_ = 0.44, *p* = 0.67) between responders (*n* = 12) and nonresponders (*n* = 8).

#### 3.1.6. White Matter Tractography for the SMc cTBS Group

**Figure 7:**
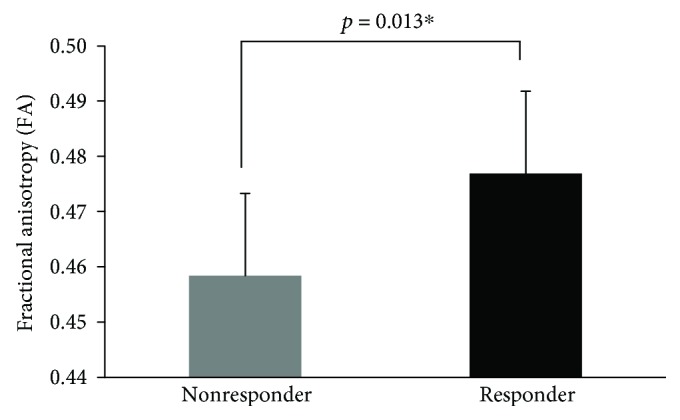
Comparison (mean [SD]) of responder versus nonresponder of CMC in the SM-cTBS group. Error bars represent SD.

**Figure 8:**
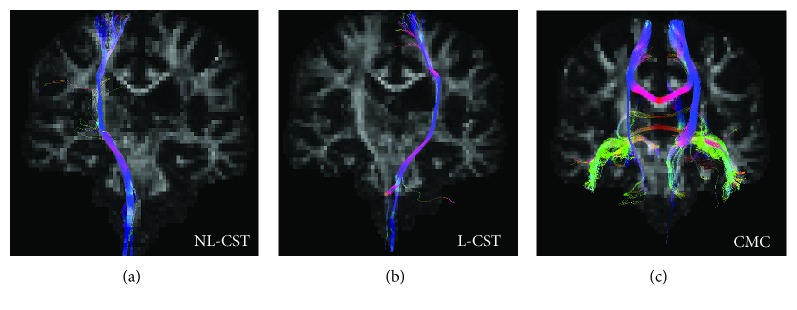
Subject-specific examples of white matter tractography. Fractional anisotropy (FA) and overlaid red-green-blue-colored FA tractography: red (left/right), green (anterior/posterior), and blue (superior/inferior). Examples of DW-WM tracts from the (a) nonlesioned corticospinal tract (NL-CST), (b) lesioned corticospinal tract (L-CST), and (c) constrained motor connectome (CMC) for a motor practice responder in the SMc-cTBS group.

In the combined SMc-cTBS group, following the GROUP (responder, nonresponder) × WM-FA (NL-CST, L-CST, and CMC) MANOVA, there was a significant main effect of GROUP (responder, nonresponder) for WM-FA in NL- and L-CST and CMC (Wilks' *λ* = 0.62, *F*_(3, 16)_ = 3.24, *p* = 0.05, *η*_*ρ*_^2^ = 0.38). *Post hoc* univariate tests revealed that WM-FA from tracts within the CMC (*F*_(1, 18)_ = 7.69, *p* = 0.013; see Table 4, Figure 7) were significantly higher (greater linear diffusion) in responders (CMC-FA: *M* = 0.48, SD = 0.0149) compared to nonresponders (CMC-FA: *M* = 0.46, SD = 0.0113). However, FA from the NL- and L-CST did not significantly differ between responders and nonresponders (*F*_(1, 18)_ ≤ 3.34, *p* = 0.084; see Table 4). Therefore, group differences in the microstructural integrity of the CMC network had higher predictive value than CST tracts (see Figure 8 for subject-specific examples of white matter tractography).

## 4. Discussion

We demonstrated that the residual white matter integrity of the CMC was significantly different between motor practice responders and nonresponders to contralesional cTBS paired with skilled motor practice. We showed that independent of receiving cTBS over the (1) contralesional primary motor cortex (M1c), (2) contralesional primary somatosensory cortex (S1), or (3) sham stimulation, individuals with chronic stroke demonstrated the ability to learn a motor sequence. This was supported by improved performance of both the repeated and random sequences at retention and lower RTTs for the repeated versus random sequence.

The lack of behavioural differences across stimulation groups is consistent with variable interindividual responses to noninvasive brain stimulation observed in previous studies [2, 7]. This finding motivated our investigation into a subgroup of “responders.” In the current work, motor practice responders who received cTBS (regardless of stimulation site) showed differences in RTT in comparison to data from individuals who underwent sham stimulation. However, similar to our past work [3], there was no motor learning-related difference between the two stimulation groups (see also [3, 13, 33]). This led us to combine the stimulation group and test whether a biomarker could be used to identify who would respond to cTBS paired with skilled motor practice. We discovered that the diffusivity properties of a network that has been previously identified as important for motor learning in healthy older adults, the CMC, also differed between responders and nonresponders.

Our finding, that a complex WM motor network (the CMC) is related to the responsiveness of individuals to cTBS paired with motor practice, extends previous findings, showing that greater anisotropy of white matter tracts is important in stroke recovery [1, 2, 34–36]. Similar to previous studies, responsiveness to noninvasive brain stimulation was not explained by standard demographics, such as age, or stroke severity or paretic arm motor function [2, 7]. Contrary to prior literature, poststroke duration [37, 38], stroke location [39, 40], and corticospinal tract integrity [2, 7] did not characterize responsiveness to cTBS paired with motor practice. Inconsistency in measures that explain variability in response to noninvasive brain stimulation may reflect the lack of generalization between stimulation protocols (i.e., continuous versus intermittent TBS; brain region-stimulated [M1 versus S1]; contralesional versus ipsilesional hemisphere) [41]. To further the field of rTMS and poststroke recovery, future work is needed to define the specific impact of varying stimulation parameters and sites.

Following stroke, spared bihemispheric neuronal connections between direct pathways of the M1 and the CST, as well as indirect pathways such as the reticulospinal and/or rubrospinal, may contribute to positive capacity for motor change [8, 9, 42, 43]. Given the bihemispheric representation within the CMC, our findings may reflect the overlap between pathways in the CMC and those involved in interhemispheric signaling during cTBS stimulation and motor learning.

Our methodological approach for evaluating motor performance and stratifying responders and nonresponders was closely based on previous segregation procedures [2]. We employed a curve-fitting technique to categorize motor learning–related change from individual data across the entire practice period [17, 19]. Assessment of each individual's capacity for motor learning–related change in this manner is not constrained to a predetermined set number of trials, but is based on the curvilinear pattern of performance change. As the field of stroke rehabilitation works to identify biomarkers, curve fitting presents a refined method for capturing behavioural states that could be applied to other interventions [44]. In the motor practice responder group (positive *B* score), there was a significant difference when comparing SM-cTBS stimulation with sham stimulation in change in response time. There was no difference between cTBS groups (M1c, S1c). Improvements in the performance of complex motor skills involve broad networks and strengthened connections between the sensory and motor cortices [3]. Shorter response times across practice may reflect enhancements in the encoding processes for force, target direction, and egocentric coordinate transformations that occur between motor and sensory cortices [3]. The interaction between M1 and S1 cortices during skill learning is critical, and our behavioural findings may reflect the reciprocal strengthening of connections in individuals with undisrupted WM linkage between regions. Alternately, our findings may indicate that individuals with a more intact motor network at baseline have a greater capacity for motor recovery. Rather than showing an isolated effect of cTBS on M1 versus S1, our data suggest that in individuals with a greater degree of WM integrity after stroke, it is likely that a broad network of regions responds to cTBS to promote motor learning.

### 4.1. Limitations

The identification of specific biomarkers that distinguish responders from nonresponders is an important first step in understanding the mechanisms of action of noninvasive brain stimulation paired with motor practice [45]. A limitation of our study is the relatively small sample size (*n* = 28; M1c = 9, S1c = 11, and sham = 8). A larger sample may help to verify the CMC as a biomarker of cTBS response. Beyond our planned comparisons (two-tailed and one-tailed independent *t*-test), we observed a lack of behavioural effects and interactions between groups and sequences (random, repeated) using inferential statistics for performance of the serial tracking task. Furthermore, it is important to consider that the *B* value, which measures the expected change score, and was used to differentiate between motor practice responders and nonresponders, may be indicative of poor and good early performance, respectively. The motor skill nonresponders may have performed the task faster earlier in practice and therefore demonstrated a ceiling effect and less improvement in performance over the 5 days. While additional independent *t*-tests demonstrated that there was no statistical difference between the SM-cTBS motor practice responders and nonresponders for repeated sequence mean RTT at baseline, the group means showed motor practice responders had worse baseline performance (*M* = 16.07, SD = 6.25) compared to the motor skill nonresponders (*M* = 12.70, SD = 6.81). In addition, the predicted asymptote value, *A*, which reflects estimated plateau in performance, was not statistically different between motor practice responders and nonresponders, demonstrating similar practice-end motor performance levels. However, future research should investigate the response to cTBS over the contralesional hemisphere paired with motor practice in a more homogeneously impaired group of individuals with stroke.

An important alternative interpretation of our findings is that changes in motor performance may have been related to the preexisting white matter microstructural characteristics as opposed to a cTBS effect. With a larger sample, further comparisons between M1c and S1c cTBS and sham groups are needed to determine the underlying factor of change. In the current paper, we performed exploratory correlational analyses to evaluate the relationships between DWI data (CMC, NL-, and L-CST FA values) and exponential change score (*B* value). This was done in two ways: one using data from individuals with stroke in the SM-cTBS group and the second using data from the entire group (SM-cTBS, sham groups). Only CMC white matter integrity showed a relationship with motor learning (*B* score) in the SM-cTBS group. This relationship was not observed for the group when individuals in the sham condition were included. These findings illustrate that greater integrity of the preexisting white matter microstructural of the CMC appears an important factor to drive larger change in motor performance when cTBS is applied over M1c/S1c and paired with motor skill practice, compared to motor skill practice alone.

The *B* value we calculated was based on the results of sessions 1 to 7; however, there were two missing data points for two participants in the sham group. We fit the data to the performance curve and calculated the *B* value with the missing data points omitted. Yet, performance curves are a robust method to capture the overall trend in performance data over time; curves are less susceptible to outliers, missing data, and random fluctuations than calculating the overall mean. Therefore, we believe utilizing curves to be an appropriate method to determine the capacity for performance change when missing data points exist; however, we acknowledge that missing data could bias the present findings and minimize the accuracy of predicting the trend in the data.

Finally, the CMC is a group-level approach; the diffusion-weighted images are normalized to MNI space to overlay a common motor network mask. Ideally, individualized masks created from an fMRI motor learning experiment prior to receiving a noninvasive brain stimulation intervention, in combination with the CMC, may help predict individualized responses to cTBS paired with skilled motor practice. Nevertheless, past work has shown that masks generated in native versus standard space do not yield significantly different information pertaining to WM microstructural integrity [46]. Our findings will support future work to investigate the possible usefulness of using fMRI-guided DWI as a methodological approach to identify biomarkers of recovery.

### 4.2. Future Studies

Individuals with stroke develop compensatory patterns of activation that promote rapid changes in motor function [47]. However, these compensatory patterns of activity may have long-term detrimental effects [48] that are independent of improvements in motor impairment. Shifting individuals into a more normal pattern of activation early post-stroke may be an important mechanism for long-term motor recovery. As such, the capacity to determine responder and nonresponder biomarker profiles for noninvasive brain stimulation protocols is an important field of inquiry. Future studies need to determine individual functional and structural connectivity patterns associated with changes in motor function that evolve naturally (sham stimulation) compared to changes induced via noninvasive brain stimulation. Serial imaging of fMRI and DTI connectivity has been suggested as means for determining the relationship between behavioural and brain changes; however, many studies only examine changes pre- and post-intervention [44] and consider individuals in the chronic phase of recovery. Formulating experimental designs to investigate individual differences *throughout* interventions is essential to the understanding of variations in outcome measures and, furthermore, is central to derive maximal individualized treatment effects. Indeed, not all individuals demonstrate improvements over the same planned trajectory or number of practice sessions practice [17, 49]. Thus, individualized interventions are needed, based on persons' own potential for improvement.

## 5. Conclusions

The residual WM structure of a novel motor network in the brain, in the chronic phase of stroke, has emerged as a potential biomarker of motor recovery. The underlying neurophysiological mechanisms that yield the relationship between WM pathways and response to repetitive noninvasive brain stimulation needs further investigation. Findings from repetitive noninvasive brain stimulation studies have resulted in positive outcomes in stroke populations [50, 51]. However, the effects of noninvasive brain stimulation are known to be variable, which suggests that there are specific underlying mechanisms that drive activity-dependent plasticity following noninvasive brain stimulation paired with motor practice [51]. The findings from the present study demonstrate the potential importance of evaluating widespread, functionally relevant WM networks to characterize response profiles of individuals with stroke.

## Figures and Tables

**Table 1:** Participant characteristics.

Participant	Stimulation group (M1c = 1; S1c = 2; sham = 3)	Lesion location (C = cortical; SC = subcortical)	MOCA	UE FM^a^	PSD^b^	Age
ST1	1	C	28.0	55.0	270.0	59.0
ST2	1	SC	26.0	63.0	37.0	50.0
ST3	1	SC	30.0	62.0	67.0	65.0
ST4	1	SC	26.0	56.0	94.0	64.0
ST5	1	SC	26.0	59.0	12.0	82.0
ST6	1	SC	23.0	41.0	196.0	46.0
ST7	1	SC	26.0	62.0	20.0	62.0
ST8	1	SC	26.0	16.0	22.0	57.0
ST9	1	SC	25.0	30.0	160.0	57.0
ST10	2	SC	25.0	59.0	82.0	67.0
ST11	2	C	27.0	60.0	142.0	73.0
ST12	2	SC	27.0	56.0	83.0	71.0
ST13	2	C	25.0	60.0	35.0	85.0
ST14	2	SC	29.0	62.0	81.0	76.0
ST15	2	SC	29.0	54.0	23.0	60.0
ST16	2	SC	24.0	7.0	94.0	57.0
ST17	2	C	21.0	62.0	24.0	55.0
ST18	2	SC	23.0	11.0	36.0	93.0
ST19	2	C	29.0	18.0	33.0	33.0
ST20	2	C	27.0	62.0	31.0	69.0
ST21	3	SC	28.0	23.0	41.0	63.0
ST22	3	SC	30.0	35.0	27.0	56.0
ST23	3	SC	28.0	58.0	20.0	71.0
ST24	3	SC	26.0	49.0	155.0	76.0
ST25	3	C	28.0	57.0	15.0	69.0
ST26	3	SC	24.0	61.0	18.0	79.0
ST27	3	SC	26.0	29.0	47.0	51.0
ST28	3	SC	21.0	57.0	27.0	83.0

C = cortical; M1c = contralesional primary motor cortex; PSD = poststroke duration; S1c = contralesional primary somatosensory cortex; SC = subcortical; UE FM = upper-extremity Fugl Meyer.

**Table 2:** Constrained motor connectome.

CMC	MNI coordinates (*X* *Y* *Z*)	mm^2^
Right postcentral gyrus	36 −28 70	4171
Left cerebellum (V)	−16 −52 −22	704
Left superior parietal lobule	−32 −56 62	124
Right cerebellum (VI)	−26 −60 −26	114

The areas of the motor learning network were used as regions of interest, overlaid on diffusion-weighted images prior to tractography.

**Table 3:** Participant characteristics for motor practice responders and nonresponders in M1c, S1c, and sham groups.

Group	Stim group	Age (yr)		Stroke location (C, SC)	PSD (months)		UE FM		Paretic WMFT rate	
		Mean	SD		Mean	SD	Mean	SD	Mean	SD
Responders *n* = 17	M1c = 5	66.3	14.41	C = 5	75.8	73.45	46.8	20.08	38.7	17.42
	S1c = 7			SC = 12						
	Sham = 5									
Nonresponders *n* = 11	M1c = 4	63.7	11.34	C = 2	54.7	45.43	49.1	20.11	40.2	19.12
	S1c = 4			SC = 9						
	Sham = 3									

C = cortical; M1c = contralesional primary motor cortex; PSD = poststroke duration; S1c = contralesional primary somatosensory cortex; SC = subcortical; UE FM = upper-extremity Fugl Meyer; WMFT = Wolf Motor Function Test.

**Table 4:** Comparison (mean and SD) of responder versus nonresponder DWI characteristics in the SMc-cTBS group.

DWI	Responders		Nonresponders		*F* test df _(1,18)_	*p* value
	Mean	SD	Mean	SD		
NL-CST	0.50	0.02	0.48	0.04	3.34	0.084
L-CST	0.42	0.08	0.46	0.05	0.94	0.345
CMC	0.48	0.01	0.46	0.01	7.69	0.013^∗^

Significant effect of DW-FA between groups: Wilks' lambda = 0.62, *F*_(3, 16)_ = 3.24, *p* = 0.05. Post hoc univariate tests revealed FA from tracts of the CMC (*F*_(1, 18)_ = 7.69, *p* = 0.013) was significantly higher in responders versus nonresponders.

## Data Availability

The behavioural and MRI data used to support the findings of this study are available from the corresponding author upon request.
